# A Meta-Analysis Comparing Venoarterial (VA) Extracorporeal Membrane Oxygenation (ECMO) to Impella for Acute Right Ventricle Failure

**DOI:** 10.7759/cureus.19622

**Published:** 2021-11-16

**Authors:** Ziad R Affas, Ghaid G Touza, Saif Affas

**Affiliations:** 1 Internal Medicine, Henry Ford Macomb Hospital, Clinton Township, USA; 2 Internal Medicine, Hawler Medical University, Erbil, IRQ; 3 Internal Medicine, Ascension Providence Hospital, Detroit, USA

**Keywords:** va ecmo, impella, mechanical circulatory support, rv failure, acute right ventricular failure

## Abstract

The right ventricular complication happens when the right ventricle (RV) fails to move sufficient blood through the pulmonary circle to enable enough left ventricular pumping. A significant pulmonary embolism/right-sided myocardial infarction may cause this to develop suddenly in a previously healthy heart, but many of the patients treated in the critical care unit have gradual, compensated RV failure as a result of chronic heart and lung disease. RV failure management aims to decrease afterload and improve right-side filling pressures. Vasoactive medications have a lower effect on lowering vascular obstruction in the pulmonary circulation than in the systemic circle because the vascular tone is lower in the pulmonary circulation. Any factors that induce an elevation in pulmonary vascular tone must be addressed, and selective pulmonary vasodilators must be administered in a prescription that does not result in systemic hypotension or compromise oxygenation. The system-based systolic arterial pressure should be kept near the RV systolic pressure to ensure RV perfusion. When these efforts prove futile, judicious application of inotropic medications for better RV contractility may help ensure cardiac output. After obtaining the finest medical treatment, certain individuals may need the implantation of a mechanical circulatory support device.

This meta-analysis is intended to compare the Impella and venoarterial (VA) extracorporeal membrane oxygenation (ECMO) mechanical supports for patients with acute right ventricular failure. This comparison should demonstrate the best mechanical support between the two through thorough analysis.

The analysis was begun by data collection from relevant sites; PUBMED and EMBASE were searched in collaboration with Google Scholar. Keywords were searched: Impella for acute right ventricle failure and VA ECMO for acute right ventricle failure. The results that were close to the search titles had their respective articles downloaded for further scrutiny.

The search finally brought 1001 related articles that were exposed to further analysis to find more refined and closer articles within the needs of this meta-analysis. After extensive scrutiny, 23 articles were found to be the best for these analyses. The data showed that VA ECMO had better results than Impella for acute RV failure. However, the data were not statistically significant, as either the numbers of the studies were not enough or the null hypothesis was true and there was no true difference between them. More studies will be needed to confirm this.

## Introduction and background

Pathophysiology of acute right ventricular (RV) dysfunction

**Figure 1:**
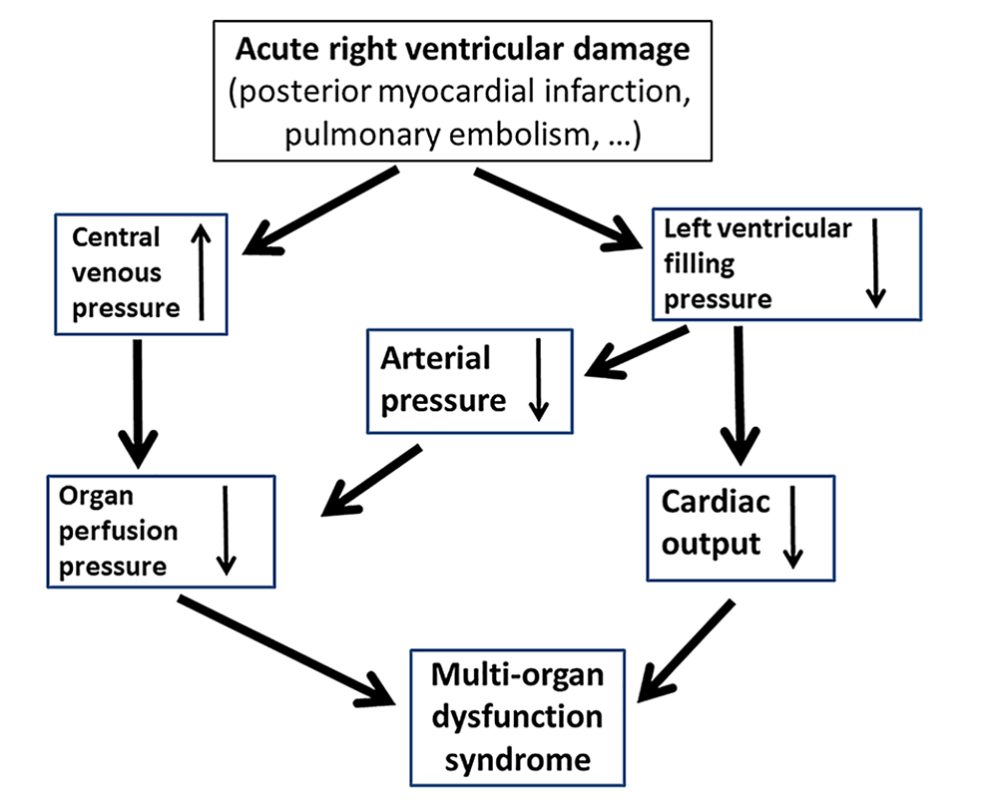
Pathophysiology of multi-organ failure secondary to acute right ventricular failure

The left ventricle (LV) and RV functions are linked together. Even while the LV stroke effort ensures blood flows forward into the stiffer high-pressure channels, the RV makes relatively little change in the low-pressure circulatory vessels that feed the pulmonary arteries [1]. The right ventricle is thin-walled, compliant, and part of a low-pressure system (pulmonary arteries circulation). All this makes it able to accommodate a large amount of blood [2]. Clinical investigations have associated right ventricular inability with an expanded danger of momentary mortality following myocardial localized necrosis. Intense right ventricular myocardial dead tissue happens in 33% of all cardiovascular failures [3]. A pneumonic embolism is the second most normal reason for the abrupt right cardiovascular breakdown. Myocarditis, intense ongoing aspiratory hypertension, postcardiotomy condition, pericarditis with pericardial emanation, cardiomyopathy, including right ventricular dysplasia, left ventricular circulatory help gadgets, and heart transfers are among the other potential causes [4]. In congestive cardiovascular breakdown, right ventricular disappointment diminishes heart yield (cardiac output or CO) and builds focal venous strain (CVP) because of the deficient filling of the left ventricle. Organ perfusion pressure is the distinction between mean blood vessel pressure (MAP) and focal venous strain (CVP). A decrease in MAP prompted by diminished CO combined with a lower CVP causes multi-organ hypoperfusion disorder and extreme cardiogenic shock. If the patient is to live and recover completely, organ perfusion pressure must be rapidly restored and maintained. With prompt circulatory support, this hazardous hemodynamic situation may be addressed [2]. Recent animal studies have shown that pulsatile support is helpful for right ventricle recovery. Pulsatile circulatory support is preferable to persistent flow pumps for the patient's microvasculature and coagulation state. Pulsatile supports, for example, the intra-aortic balloon pump (IABP) have not reduced overall mortality in cardiogenic shock since the circulatory support is so low (0.5 liters/min). Individuals with advanced heart failure have a higher mortality risk when right ventricular pulsatility is impaired. As a result, pulsatile support in acute right heart failure may be beneficial and may hasten recovery [5]. This opens up a novel therapeutic option for preserving hemodynamic stability and avoiding the development of multi-organ failure syndrome in extreme myocardial infarction with RV association or acute pulmonary embolism. That's why our team set out to find an ultra-compact right ventricular assist device that might help patients with extreme RV failure with pump flows of more than 3 liters per minute maintain hemodynamic stability. See Figure 1 for the pathophysiology.

Venoarterial (VA) Extracorporeal Membrane Oxygenation (ECMO)

**Figure 2:**
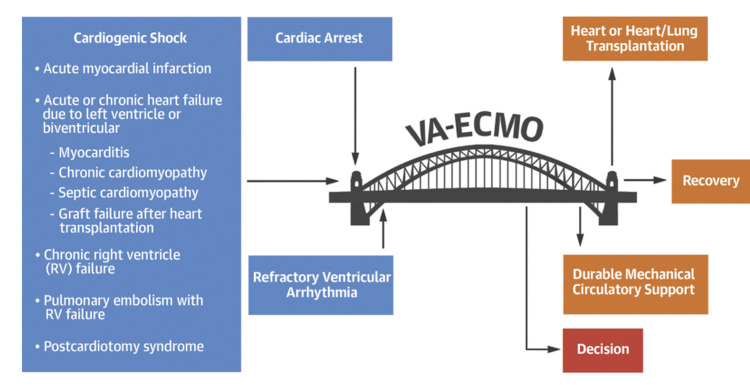
VA ECMO Extracorporeal membrane oxygenation (ECMO) works like a bridge linking patients with cardiac failure to recovery. VA: venoarterial

VA ECMO is a kind of cardiopulmonary passage that has been modified to allow patients to move about. It may be used to keep critically sick patients with chronic cardiopulmonary failure alive for many days or even several weeks, depending on their condition. ECMO has been mainly utilized in neonatology for more than 40 years, even though it is available in other areas of medicine. Patients with excess but treatable lung diseases, such as influenza, pneumonia, and adult respiratory distress syndrome, were the primary beneficiaries of venovenous (VV) ECMO when it was initially introduced into clinical practice [6]. Over the last decade or two, there has been a rise in the use of this technology in the field of adult cardiology. Extracorporeal cardiopulmonary resuscitation (ECPR) is superior to conventional cardiopulmonary resuscitation in terms of neurologically intact survival in studies involving prospectively observed propensity-matching research of adult VA ECMO. ECMO circuits extract deoxygenated blood from the venous system, which is then pumped through an oxygenator for gas exchange. One or more drainage cannulae are used in this procedure. A reinfusion cannula is used to reintroduce the blood back into the venous or arterial circulation after it has been removed. ECMO works like a bridge linking patients with cardiac failure to recovery [7]. Figure 2 depicts the VA ECMO process.

**Figure 3:**
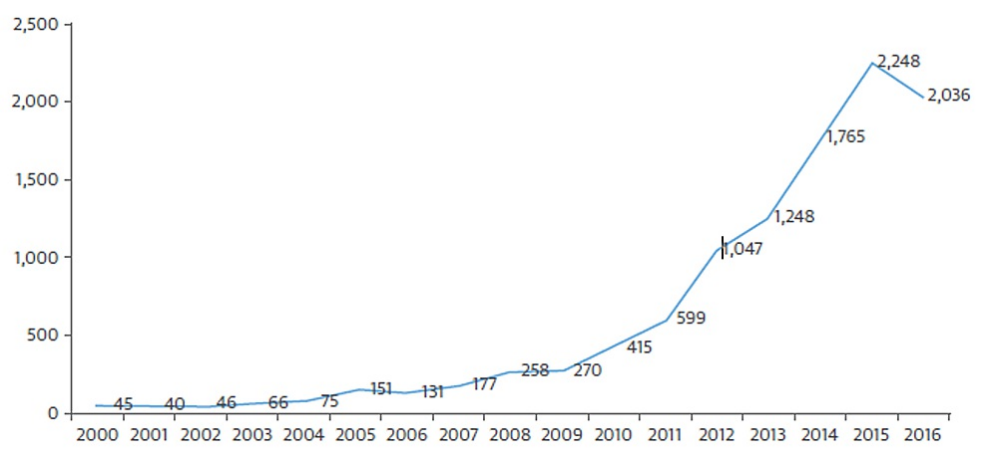
Numbers of adult cardiac ECOM Data from the Extracorporeal Life Support Organization

Increasing numbers of adult cardiac ECMO oxygenation procedures are being performed. In the past 10 years, there has been a 1,180 percent rise in the amount of money available. Between 1997 and 2007, there were about 200 runs per year. Each year between 2014 and 2016, there were almost 2,000 runs total. Data from the Extracorporeal Life Support Organization was used to compile the research that was discussed in this article [6] (Figure 3).

Impella

There has been a considerable rise in the usage of subcutaneous mechanical support (SMS) in the past 10 years. Following recent findings on the dubious usefulness of intra-aortic balloon pumps (IABPs), especially in sensitive post-infarction shock, it is expected that emerging devices that provide better circulatory support would have more therapeutic benefits [8-9]. The TandemHeart or ECMO and the Impella transaortic intraventricular pump [8] are among the most commonly utilized extracorporeal devices. The Impella device has been authorized in Europe since 2005, according to the European Commission, for a range of manifestations, with the inclusion of high-risk percutaneous coronary intervention (PCI) and cardiogenic shock. It was also legalized in 2006 in Canada, 2008 in Latin America, and 2013 in China, to name a few. Around 8000 patients are believed to have received assistance from sources outside of the United States in the past eight years [10]. In the United States, there are approximately 800 hospitals that care for more than 20,000 patients [10]. There are significant differences across nations when it comes to indications for usage and the specific devices that are utilized. Uncertainty over the best use of particular devices in different clinical situations, as well as disparities in reimbursement conditions throughout the area, may all play a role [11]. The Impella Working Group, which has a combined exposure of over 1000 European Impella implantations, seeks to provide a current overview of the state of the art, allowing for more in-depth knowledge of the Impella technology's foundations and fundamental application [8]. The Impella system consists of a 9 F catheter and a small axial pump that work together. Blood may be taken from the left ventricle and then released via the aortic valve into the ascending aorta through the cannula part, enabling the heart to operate properly. The pump motor and sensor connections are powered by the power connectors on the 9 F guiding catheter. The catheter's opposite end is linked to an external console that includes a pump and a purging system controller. Impella catheters are varying in terms of size, insertion method, and maximum flow capacity.

Discussion on Impella

Impella Access Site Management

To avoid the difficulties associated with bigger sheaths, the Impella support access point should be carefully selected and maintained. When deciding on the site access and implantation method for an elective Impella implantation, consider the patient's anatomy as well as the operator's level of expertise. The Impella 2.5 and CP should be implanted percutaneously via the common femoral artery while Impella 5.0 should be implanted through the axillary artery (through a surgical procedure). An angiography may be used to determine whether or not arterial access is required in a small period. Even though Impella may be treated with closure devices like Perclose (Abbott Laboratories, Abbott Park, Ill) for many days, longer-term implants should not be cured using percutaneous preclosure methods owing to the risk of infection. By removing the peel-away sheath and using the repositioning sheath, the arterial flow interruption caused by the Impella catheter may be prevented [12]. In the overwhelming majority of cases, manual compression is adequate; nevertheless, surgical closure should be considered in certain situations. Vascular insufficiency is an uncommon disease that must be thoroughly examined and treated as soon as possible. When appropriate access control measures are used with Impella support, there is a minimal chance of severe arterial problems (similar to IABP) [13-14].

Impella Placement and Position During Long-Term Support

Proper Impella posture is required at all times while providing long-term support for the Impella product line. The device must be correctly positioned over the aortic valve to prevent complications such as suction episodes, hemolysis, and insufficient hemodynamic support. To begin paying attention to devise placement in the catheterization lab or operating room, the first step is to avoid migration into the left ventricular (LV) chamber [14]. Excess slack from the catheter in the ascending or transverse aortic arch is the most effective way of accomplishing this goal. Excess slack is eliminated from the catheter when the Impella is set to high thrust condition, allowing it to lay along the inner curvature of the aorta and the positioning marker to be roughly near the aortic valve [12]. As soon as it is finished, the intake should be about 3.5 cm away from the aortic valve. Make sure not to entrain the catheter in the papillary muscles or mishandle the anterior mitral leaflet while you are inserting the catheter. Subannular sites should be avoided if at all possible [10]. Because patients move often, it is important to pay close attention when positioning warnings and possible position loss are shown to the caregiver. A significant collaborative effort between cardiologists who are acquainted with percutaneous circulatory support and intensive care unit doctors who are trained in both the ICU and the Cardiovascular Care Unit is needed to guarantee appropriate placement after a patient transfer.

Position and Operation of Impella in RV

Impella RP is a percutaneous mechanical circulatory support device that allows for early intervention in medically resistant right ventricular failure (RVF). A prompt introduction of mechanical assistance may act as a bridge to recovery, a heart transplant, or destination treatment in the right RVD given the reversible nature of the disease [13]. Since the pump's input is located in the inferior vena cava (IVC) and its outflow in the pulmonary artery (PA), the pump may move up to 4 liters of blood per minute between the IVC and PA. The tip of the Impella RP (Abiomed, Danvers, MA) should be positioned in the major pulmonary artery, with the catheter in the right pulmonary artery, to properly monitor RV function [15].

One of the first studies to show that the Impella RP is hemodynamically successful included 30 participants in the RECOVER RIGHT trial [12]. It was shown that 18 people had RVF within 48 hours of having a left ventricular assist device (LVAD) implanted and that 12 people developed RVF within 48 hours after having cardiac surgery or having a heart attack in the study [12]. When Impella RP support was started, the cardiac index decreased immediately, and central venous pressure was reduced as well, leading to better outcomes and a survival rate of 73.3% after 30 days. In a second Impella RP pre-market clinical trial, including 60 patients with RVF, the therapeutic advantage of Impella RP was proven once again [16]. One post-approval study [13-14] included 42 patients with RVF and found that the 30-day survival rate was 64% for those with the RECOVER RIGHT inclusion and only 11% for those with Impella RP used as salvage support in the patients who had RVF at the time of the study's completion. With Impella RP's poor results in salvage patients [15], it's clear that appropriate patient selection and early start of hemodynamic support with Impella RP is required for success. 

Patient Selection

When the conditions allow, pre-procedural imaging of the RV to rule out the existence of a thrombus is highly recommended. When an Impella ingests a blood clot in the RV, it is quite probable that the Impella will cease working altogether. Regardless of how careful you are, there is always the risk of inadvertently harming yourself or someone else if you swallow some of the catheter's contents by mistake. Severe aortic regurgitation is another contraindication to this procedure (AR). The installation of a reliable valve to separate the left ventricle from the aorta enables Impella-calculated forward flow to be as efficient as possible [14]. According to the American Heart Association, patients with decompensating hemodynamics caused by AR will be underserved by Impella support since raising aortic pressure may exacerbate AR and increase aortic and RV dilatation.

Acute right ventricular failure without hemodynamic support is shown in blue in the pressure-volume loop; Impella CP (Abiomed) support is depicted in green, and ECMO support is depicted in yellow (red). It is necessary to know the loop area to compute the mechanical effort of the ventricle. Pay close attention to how much space is saved by the Impella device, as well as the distinct oblique vertical lines indicating that the ventricle is continuously drained, even during the "isovolumic" stages of the procedure [15].

High-Risk Intervention

Patients who have been denied coronary course sidestep joining (coronary artery bypass graft, CABG) because of an unsuitable danger of inconveniences or passing might have one more revascularization choice as percutaneous coronary intercession (PCI). There are numerous potential clarifications for this. Patients who go through PCI, are again in danger of hemodynamic breakdown, conceivably deadly musicality anomalies, and peri-procedural sequelae. Then again, if circulatory help is started before mediation, it very well might be feasible to do complex PCI without encountering fast circulatory decay during coronary impediment, considering broader reperfusion treatment. The intricacy of coronary course infection, just as clinical co-morbidities, including old age, diabetes, and renal brokenness, just as an earlier technique history, decide whether a patient has a high-hazard clinical show [17]. Confounded treatments frequently need long treatment times, complex strategies, for example, rotational atherectomy, and a higher danger of intense blood vessel block, low-stream or far-off embolization, and heart corruption. These issues may be kept away from if easier strategies were utilized. You might have to play out a ton of stenting to get the best results. After the primary endpoint of multi-day major adverse cardiovascular events (MACE), there was no huge contrast between the Impella and control gatherings, but at 90 days, the Impella bunch showed a generous decrease in MACE. In a randomized controlled review that was rashly ended, the utility of Impella help with high-hazard techniques was examined. Impella backing might be a more savvy treatment decision in the United States and the European Union than IABP counterpulsation [13]. Notwithstanding the constraints of the current information, our agreement board knows from clinical experience that specific patients advantage from mechanical peri-procedural help in arranged high-hazard PCI. Regardless of whether there aren't any unmistakable guidelines, it's fundamental to assess the dangers versus benefits [15].

Management of Impella

Anticoagulation

To give Impella support, two anticoagulant arrangements should be utilized independently and independently: 1. Foundational anticoagulation titrated utilizing ordinary strategies; 2. Anticoagulant infused to the cleanse liquid to forestall blood passage into the engine chamber and pushed out into the plunging aorta and the fundamental UFH might be quit, empowering the ACT to diminish into the 150 s range. Long-term support frequencies are provided. A very low incidence of complications occurs with elective high-risk operations, and this is similar to the incidence seen with IABP. N40 mg/dL hemoglobin free [17].

Echocardiography

Transthoracic (TTE) or transesophageal echocardiography (TEE) bedside echocardiography should be 24 hours available a day, seven days a week (TEE). Images taken with TTE give the finest look in the parasternal long-axis perspective. In this case, it's critical to locate the device's intake, which should be approximately 3.5 cm from the RV cavity's vena cava valve. The best TEE research is typically obtained at the mid-esophageal view with a rotation of 120-130 degrees [13]. Because the aortic "slack" may not always translate into the proper pump movement, continuous echocardiographic guidance should be utilized while moving the catheter. Because of turbulence and color aliasing, color flow mapping is difficult to comprehend, but when Impella is properly positioned, the "mosaic" of colors associated with the device's outflow should appear above the aortic valve [16]. If there is any suspicion that the device has been misplaced, echocardiography should be performed in addition to left ventricular function.

Weaning

As long as the patient's hemodynamics remain stable, no further pharmacological inotropic assistance is needed. The results of short weaning trials performed under echocardiographic supervision may be used to determine if myocardial healing has occurred. Weaning off Impella is accomplished gradually over the last four to six hours, with support decreasing to about 1-1.5 L/min over the last four to six hours [18]. The use of echocardiography is utilized to confirm the recovery of left ventricular function and the long-term stability of the cardiac index. To ensure that the baby's SVO2 (mixed venous oxygen saturation), lactate, and arterial pH levels are supervised during the same period [16]. If explantation is required, the catheter may be reinserted into the downing aorta and the systemic unfractionated heparin (UFH) can be stopped, which will enable the activated clotting time (ACT) to drop to the 150-s range again. Manual compression for hemostasis (for at least 30 minutes) may be performed after the impeller has been switched off and withdrawn after 30 minutes [10].

Common console complications

The most frequent complication is bleeding, particularly with extended usage. However, for some people, the advantages exceed the dangers [16].

Discussion on VA ECMO

Activation, Cannulation, Management, and Weaning Are All Part of the VA Ecmo Logistical Process

Each phase of an ECMO patient needs a unique set of abilities to fulfill the logistics. The experts engaged in an ECMO patient's treatment frequently depend on experience [6]. The team usually has interventional and clinical cardiologists, perfusionists, vascular or cardiothoracic operation managers, highly educated sensitive care nurses, emergency doctors, and palliative care experts. Weaning and decannulation are the first five stages.

Activation

Activation of the ECMO team, unless in an urgent matter, requires an initial interactive discourse between the members of the team to assess the possibility of success based on the clinical setting, place’s capabilities, and perfect cure projections [6]. If most of the team members consensually accept to proceed, the inserting physician will need to organize the operation with the laboratory nurses belonging to perfusionists or catheterization, as well as make preparations for a severe care bed and appropriate paramedic transport unit, if necessary.

Cannulation

Cannulation cannot be emphasized enough. Percutaneous access is needed for ECPR at the catheterization lab or the patient's bedside. In less urgent cases, surgical amputation may be considered. An anticoagulated patient's large-bore cannula must be carefully placed into an artery to avoid hematoma, bleeding, and vascular damage. Percutaneous insertion is frequently done using ultrasound guidance and micropuncture needles. Experts in the field, such as interventional cardiologists or cardiothoracic and vascular surgeons, should perform calculations. The current literature does not define the size of cannulas required for VA ECMO support. Cannulas are selected to give full support for a patient, resulting in a 2.2 l/min/m^2^ index. The pressure drop between the venous cannula and the artery side of the circuit should not exceed 100 and 300 mmHg, respectively [19]. A flow of 60 cm^3^/kg/min is required to keep an adult alive. Each cannula's flow characteristics are indicated by a pressure-flow curve in addition to its length and diameter. The UM number (flow at a pressure drop of 100 mmHg across the cannula) describes the cannula and flow characteristics anywhere from the periphery to the heart [20]. Although peripheral insertion is quicker, the huge size of the cannula has been associated with lower extremity vascular impairment, especially in small women or those with "clamped down" blood arteries. It is possible for limb ischemia when the vasculature is not 1-2 mm larger than the cannula. When a 6-F to 8-F introducer or sheath is inserted in the superficial femoral artery during cannulation, part of the arterial return flow may be directed down the leg (reperfusion catheter). Patients who do not need immediate VA ECMO may be candidates for "sport model" cannulation of the internal jugular (IJ) to the subclavian artery (SCA). Because non-emergent VA ECMO cannulation sites were moved from the groin to the upper body, patients may now walk more freely after their operations (Khorsandi et al. [19]). Ambulatory ECMO prevents deconditioning in patients awaiting transplantation. Special patient needs may be met using additional venous cannulas, such as the Avalon Elite (Getinge, Göteborg, Sweden) or the OriGen (OriGen Biomedical, Inc., Austin). Venoarterial-venous cannulation is often used as a second method. A case study of 21 patients who survived hybrid ECMO resuscitation showed a survival rate of 43% [21].

Management of ECMO flow

The aorta is typically filled by retrograde ECMO flow from the arterial cannula, which is usually sufficient for an ECMO flow of 50 to 70 ml/kg/min (4 to 6 l/min). As a consequence, the RV now has additional afterload to cope with. When right ventricular end-diastolic pressure (RVEDP) and pulmonary capillary wedge pressure are both high, pulmonary congestion may occur. To shunt blood from the RA to the left atrium and reduce the elevated right-sided pressures, inotropes [19] or an interatrial balloon septostomy may be required. Several ways of "venting" or emptying the RV are described in depth in the pathophysiology section. Another disadvantage of VA bypass surgery is the possibility of a negative cerebral event if particles, bubbles, or emboli are introduced into the arterial or venous circuits during the operation. Reduced lung blood flow may also contribute to the development of microvascular intrapulmonary thromboses, which can make already difficult circumstances even worse [22].

Monitoring

Each ECMO patient requires a blood vessel line and a pneumonic vein catheter. Some contemporary siphons (Maquet CardioHelp, for instance) ceaselessly screen blended venous immersion, conceivably hindering the requirement for a pneumonic supply route catheter, which is regularly used to screen blended venous immersion. While using thermodilution to decide heart yield, an obscure measure of the chilly bolus is brought into the ECMO circuit, making it prone to be mistaken. To evaluate aspiratory blood vessel tension as a substitute for left ventricular widening, a Swan-Ganz catheter ought to be set and used. Lactate estimation dependent upon the situation might be valuable [19].

Anticoagulation

Blood product interaction with the ECMO circuit's surface is reduced when patients are given systemic anticoagulants. One hour after ECMO is begun, fibrinogen and albumin attach to the biopolymer components of the circuit, triggering platelet aggregation, activation, and consumption [20]. If the coagulation system is activated, thrombocytopenia may develop, which can be severe and need platelet transfusions [22]. From one site to the next, the anticoagulant medication used and the quantity of anticoagulation monitored vary. Patients who take heparin follow a standard weight-based regimen, aiming for an activated thromboplastin time of 50 to 75 seconds (from a baseline of 1.5 to 2.5 seconds) or an anti-factor Xa concentration of 0.33 to 0.67 international units per milliliter (IU/ml). A few establishments select a functioning thickening time objective of 180 to 220 seconds on account of its bedside accessibility and quick turnaround time. Direct thrombin inhibitors, for example, parenteral bivalirudin and argatroban, are options in contrast to heparin in patients with heparin-instigated thrombocytopenia. These immediate thrombin inhibitors are utilized to test a patient's anticoagulation for initiated halfway thromboplastin lengths of 50 to 60 seconds [23].

Ventilator Management

Individuals undergoing ECMO may be able to avoid the need for a ventilator if their condition improves. Because ECMO allows for up to complete the gas exchange, a cognizant patient in cardiogenic shock may breathe spontaneously while on ECMO, regardless of whether or not their respiratory function is functioning. Ventilator support, on the other hand, maybe required for airway protection in patients who need sedation such as those who have had a cardiac arrest. In hospitals, low tidal volume ventilation (3 to 5 ml/kg) is often used to minimize lung damage and improve patient outcomes. One or more of the following protective mechanical ventilation settings: an 8/min rate, a positive end-expiratory pressure of 10 to 15 Torr, an inspired oxygen percentage of 0.40, a low tidal volume (Napp et al., 2016). Hemodialysis employs the technique of ECMO. In certain cases, continuous VV hemodialysis may be done utilizing an ECMO circuit, whereas, in others, separate vascular access is required. Hemodialysis using the conventional method is also available as an alternative treatment option.

Temperature

A heat exchanger is also included in the membrane oxygenator for further efficiency. As a result, it is possible to adjust the temperature of a patient as needed. This conceals the fact that you are suffering from a "fever" as well as the fact that you are suffering from an infection. It may be beneficial to maintain a patient's temperature below 36 F for up to 24 hours following an anoxic insult if extracorporeal membrane oxygenation is utilized [19].

Weaning

If a patient's condition gets better while on ECMO, weaning becomes the next stage. Withholding ECMO at slower rates, such as 0.5 l every six to 24 hours or even more slowly, down to nil, may be more effective than abruptly discontinuing it [24]. An ECMO flow of 1.5 l/min is needed to ensure a combined venous saturation of more than 65% plus an arterial saturation of greater than 90% for the patient to remain stable. An extension between the blood vessel and venous cannulas might be essential to isolate the patient's flow from the ECMO circuit without decannulation. It is clasped in case there are indications of decompensation, and the patient is re-upheld to the furthest reaches conceivable [20]. The most successive areas for decannulation are catheterization labs and working rooms, individually.

Complication of ECMO

Bleeding

Patients on VA ECMO often need anticoagulation, making them prone to clotting issues and bruising than the general population. Hemoglobin levels of 8 to 10 mg/dl are suggested for achieving optimum blood oxygen saturation. However, if transfusions are needed, there is a risk of desensitization and transfusion-related acute lung damage in a prospective transplant candidate (TRALI). Heparin (or a direct thrombin inhibitor) dose may be reduced or anticoagulation may be stopped to control bleeding. According to some studies, you may be able to safely stop taking your anticoagulant for up to three days without suffering any side effects if you have anticoagulation intolerance [19]. Protamine or, more often, factor-containing products are required when modifying anticoagulation is insufficient, as is the risk of heparin-induced thrombocytopenia (HIT).

Infection

Bacteremia and sepsis are the most common infectious VA ECMO consequences, and longer ECMO runs are linked with a greater incidence of infection. Within 14 days of starting ECMO, more than 53% of patients get infected. When there are infectious complications, mortality rates may rise as high as 60% of the population. A sterile approach must be used while doing cannulation, particularly if the operation is urgent or emergency in nature [24].

Throughout the studies, VA ECMO had treated 230 patients, and four have been diagnosed with acute RV failure together with cardiogenic shock [20-22,24]. Patients with PAH, severe pulmonary embolism, and impending cardiovascular collapse were all included, as was the last patient, who had Eisenmenger physiology as well as congenital heart disease. They were given VA ECMO as an immediate lifesaver. All of the patients had immediate hemodynamic and ventilatory improvements. The mean support period was 11 days, with a range of five to 16 days. Two patients (50%) were successfully weaned off ECMO and lived to be discharged from the hospital. After a multidisciplinary team debate, it was decided that the other two patients had irreparable multiple organ damage and ECMO assistance was discontinued.

## Review

Methods

Inclusion Criteria

Types of research: Because there were no publications that compared Impella to VA ECMO in advanced heart failure, this meta-analysis included all randomized controlled trials (RCTs) that separately addressed Impella and Va ECMO for acute heart failure. Cross-over trials, as well as quasi-randomized controlled trials and cluster-randomized trials, were not included.

Participants' types: All trials involving humans and animals with acute RV failure, heart failure, or both were included in this analysis. Individuals who underwent surgery for which VA ECMO or Impella was planned as a surgical therapy were excluded from this research.

Types of interventions: A comparison was made between ECMO utilizing pumps to drive venovenous and venous-arterial circuits as well as pump-free arteriovenous circuits (e.g. intermittent positive-pressure ventilation). Study results comparing ventricular assist devices to other mechanical cardiac support devices were omitted.

Language of the studies: We used English as the primary language for our search. All the articles that were included were in English. Other languages articles were excluded from the study

Exclusion Criteria

Studies that failed to satisfy the inclusion criteria were excluded.

Search Criteria for Identification of the Articles

Searched based on electronics, we searched PubMed and EMBASE. Other resources were additional databases of current studies like current controlled trials (http://www.controlled-trials.com/) and clinical trials (http:// clinicaltrials.gov/) for conferences, meetings, and abstracts.

Collecting, Analyzing, and Selecting the Studies

The titles and abstracts of all retrieved citations were evaluated against the inclusion criteria separately. Articles that fulfilled the choosing criteria and merited full-text access to collect more revelation were considered to be included.

Types of Outcome Measures

The primary outcome: This study's main goal was to see how well mechanical assistance for acute RV failure compares to other options.

Secondary outcomes: These included other goals to help us know how long a patient needs to be in the hospital, the ability to endure until being discharged, possible disabilities, bad consequences, the research authors' assessment of the quality of life based on health, and evaluations of long-term health and well-being carried out by researchers.

Results of the Search

A total of 1001 items were found, with 613 coming from PubMed and 388 from EMBASE. After removing all of the duplicate articles (710), just 291 articles remained for further screening. Following a rigorous initial screening process, four clinical trials and an economic assessment of one of those trials were found to be strong candidates for inclusion in this study. There were 268 publications whose titles or abstracts could not be eliminated, therefore, the whole papers had to be obtained and evaluated. None of them fulfilled the requirements for inclusion (excluded studies). Although LR and RT refreshed the search every day, no new results were found. Researchers searched databases, including current controlled studies (http://www.controlled-trials.com/) and clinical trials (http://clinicaltrials.gov/) for conference proceedings, meeting abstracts, and current trials. They were able to find three trials that were relevant to their investigation (ongoing studies). Because the 23 publications included in this research offered enough information to meet the analytic requirements, data had to be extracted from each of them.

Excluded articles

Of the 268 articles, we excluded eight papers without further analysis since they lacked RCTs, which we considered unethical. Studies that were published before 2014 were also excluded, with the most current papers getting the most priority in our meta-analysis, which was conducted in 2015.

**Figure 4:**
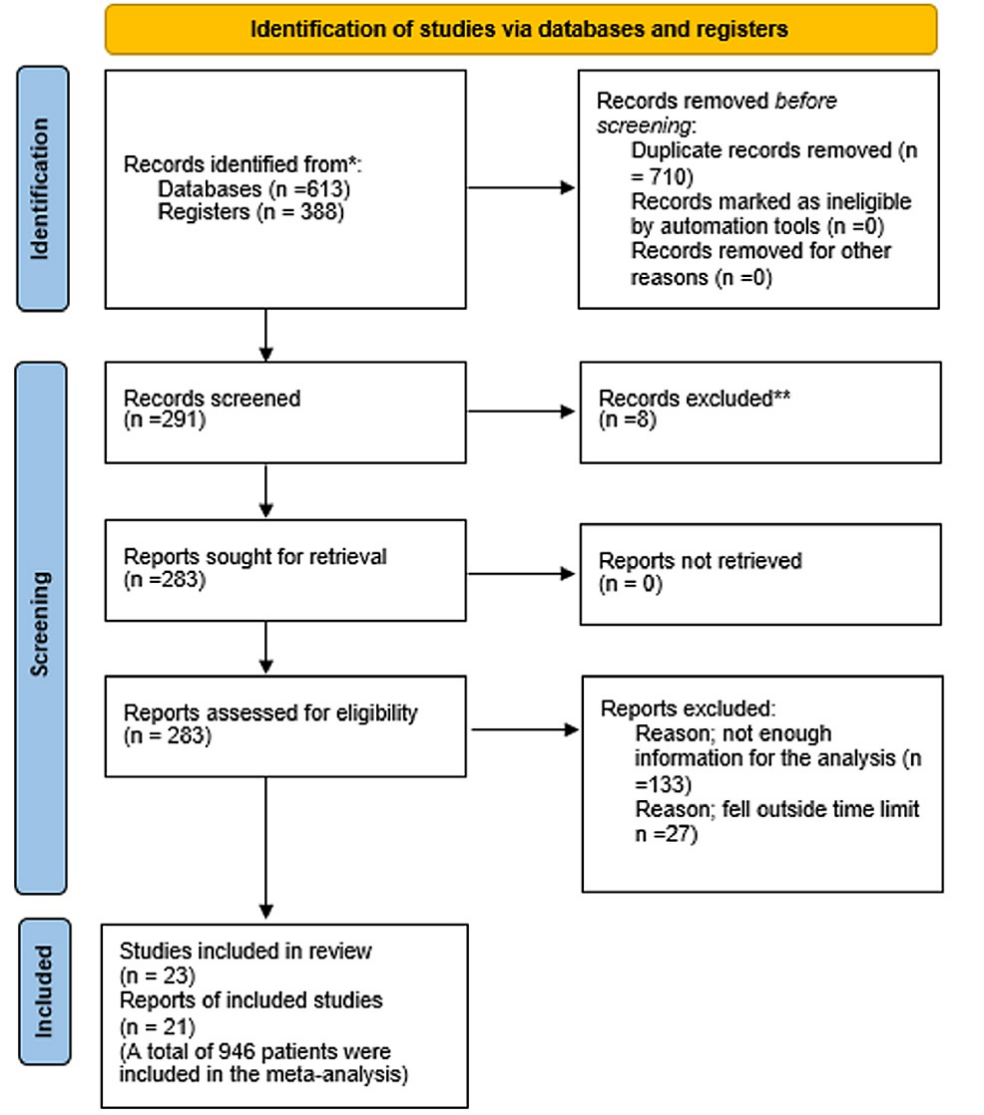
Data collection

Figure 4 shows the data collection process. 

Result of the study

**Figure 5:**
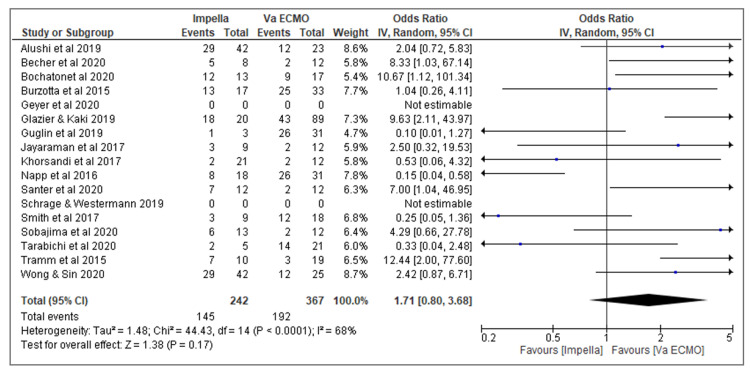
Forest plot for the study Guglin M et al. [6], Smith M et al. [7], Bochaton et al. [9] Tarabichi S. et al. [11], Burzotta F [12], Alusshi B et al. [13], Wong & Sin [14], Santer et al. [15], Geyer M et al. [16], Sobajima M et al. [17], Glazier JJ & Kaki A [18], Khorsandi et al. [19], Becher et al. [20], Tramm et al. [21], Napp et al. [22], Schrage B & Westermann D [23], and Jayaraman AL, Cormican D, Shah P, Ramakrishna H [24]

Figure 5 shows a forest plot for the study, it graphically summarizes the outcome from the studies under meta-analysis. It compares the number of patients that were treated with Impella and VA ECMO against the total for acute right ventricle failure. On the graph, the horizontal line represents each study, the line ending represents the boundaries for 95% confidence, the small box on the study reflects the number of patients in the study. The bigger the number of patients, the bigger the box and the thicker and shorter the line, indicating a more precise study and vice versa. If the small box crosses the line of null effects then the study is statistically insignificant. The numbers to the right of the graph represent the figures' visualization of the graph. The ones outside indicate the p-value for the individual study while the ones in the bracket indicate the endings of the 95% confidence intervals for every study. The diamond shape at the bottom of the graph shows the average of the entire study since the diamond shape is crossing the null effect line, the general study is statistically insignificant. For heterogeneity, the p-values are more than 0.05 as I2 is more than 50%, further indicating insignificancy. The insignificancy in this has been largely brought by a small number of patients per individual study as well as the inconsistency in the number of patients in successive studies. 

**Figure 6:**
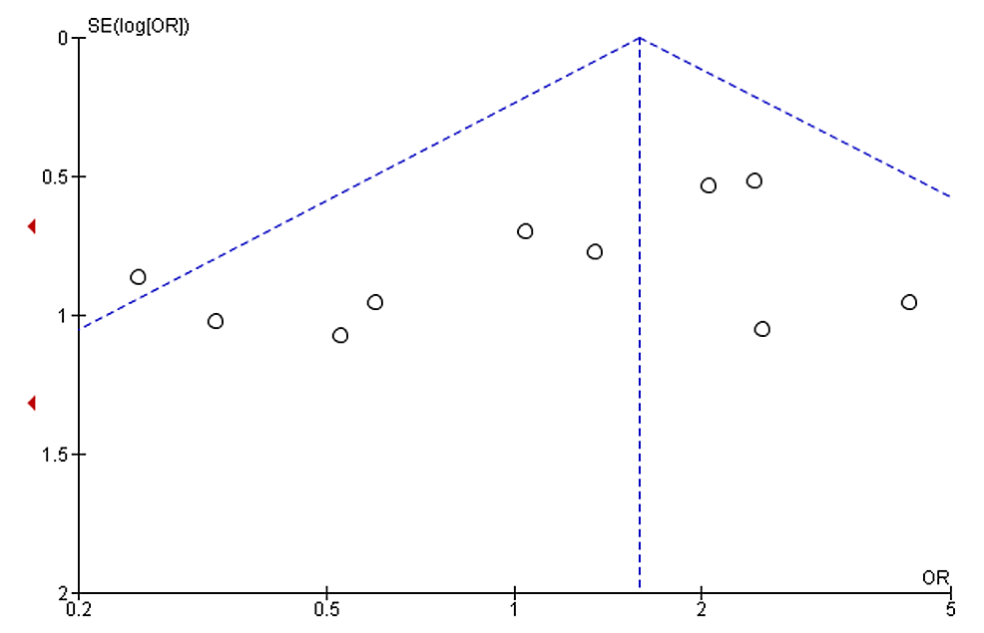
Funnel plot of the study

Figure 6 shows a funnel plot. It is used to visually indicate how precise the entire study was. In this funnel plot, the circular dot represents individual study, as the two dotted lines represent the 95% confidence intervals since the graph is nearly not symmetrical, the general study is not that price, thus indicating some bias in the general study that has been widely contributed by missing data in some of the studies as well as the variation in individual studies.

It was seen throughout the studies that the mean hospital stay for patients with Impella support ranged from eight to 16 days while that of VA ECMO ranged from 25 to 30 days. The ability of endurance before the discharge ranged from an individual to the other for both mechanical supports, some showed severe complication while others showed fair complication. The most common and serious complication of VA ECMO was bleeding, whereas bleeding and vascular problems were the most often reported side effects with Impella. Heparin, a blood thinner, is continually injected during VA ECMO, which increases the risk of bleeding. Heparin helps maintain the ECMO circuit running smoothly by preventing blood clots from forming in the vessels after an injury. The findings show that Impella offers reliable acute mechanical circulatory support for individuals who require it by making their hemodynamics stable throughout therapy and even after discharge. Among those who had VA ECMO, 40% experienced limitations on daily activities, 35% indicated mobility issues, 30% expressed anxiety/depression, and 30% reported pain/discomfort. It was shown that lower health-related quality of life (HRQL) was associated with greater levels of psychological distress, discomfort, and being unable to do the things you normally do every day. Extended time since ECMO had a substantial impact on HRQL improvement. VA ECMO use has the potential to cause developmental and neurologic abnormalities as well as other long-term issues. Low oxygen levels in the brain before ECMO or intracranial bleeding have been linked to neurological disorders.

Worth mentioning is that we found two animal studies (Josiassen et al. 2020 and Yastrebov et al. 2020) [8,10], which helped in providing introductory information to our research, however, their weight in the analysis was not estimated.

## Conclusions

In conclusion, acute right ventricle failure needs mechanical support, such as Impella and VA ECMO, for ultimate treatment, as per the analysis described above. Even though both the mechanical devices provide significant assistant to patients with this kind of heart complication, Impella proved to be relatively effective and capable of handling serious complications as the numbers of survivors were statistically high per event. VA ECMO was first used in the operating room as a cardiopulmonary bypass and has since been adapted for use in the critical care unit and beyond. Extracorporeal life support is useful in restoring circulatory support when other therapies fail in people who are anticipated to recover from the source of their RV failure or as a bridge to surgical treatments such as pulmonary endarterectomy or lung transplantation. According to a growing amount of registry and observational data, the Impella system seems to play a major role in limiting RV failure. Impella's early usage in patients with RV failure is linked with a greater probability of survival, according to new research. An Impella-supported PCI may also help with regular right cardiac catheterizations, which may enhance results. So far, there hasn't been enough RCT research to demonstrate that Impella can be used to treat cardiogenic shock. While there is a lot of evidence that Impella can help with specific high-risk PCI procedures, there haven't been any compelling RCT results to back it up. However, generally, the statistical insignificancy in the data could either mean that the null hypothesis is true and there is no difference between VA ECMO and Impella in the treatment of acute right ventricular failure or could indicate that the data could be inconclusive. Either way, more studies are required.
